# Levetirazetam: antiepileptic-induced psychosis

**DOI:** 10.1192/j.eurpsy.2022.1890

**Published:** 2022-09-01

**Authors:** C. Vilella Martín, P. García Vázquez, Y. Barrera García, P. Fernández Perea, A. Serrano García, M.Á. Alonso De La Torre López, R. Gómez Martínez, C. Franch Pato

**Affiliations:** Complejo Asistencial Universitario de León, Psychiatry, LEÓN, Spain

**Keywords:** Psychosis, levetirazetam, antiepileptic

## Abstract

**Introduction:**

Levetirazetam is an antiepileptic drug with psychiatric adverse reactions. It includes psychosis, paranoia or hallucinations. The frequency is less than 1%.

**Objectives:**

To describe a case of Psychosis produced by Levetirazetam

**Methods:**

Retrospective review of clinical records and complementary test, including psychiatry, electrophysiology and neurology. Diagnosis schales such as Salamanca Questionnaire were used as suport.

**Results:**

A 42-year-old woman diagnosed with tuberous sclerosis and undergoing treatment with levetirazetam acudes to the emergency department for behavioral disorders. She has presented an episode of aggression against a relative threatening him with a kitchen knife. The family reports that since the change in antiepilepticus 1 month ago, the patient has presented strange behaviors. Te Patient is conscious, uncooperative. Barely Approachable. Suspicious of her surroundings, with psychomotor restlessness, self-reference ideas and sparse speech. Auditory hallucinations seem to be present, as well as depressed and irritable mood. Psychic and somatic anxiety is found.

Levetirazetam is discontinued, being replaced by valproic acid. Risperidone is started at a 3 mg dose. Treatment is well tolerated, and clinical stability is achived. Cluster A personality traits are found. Complementary test Blood and Urine simples, Imaging tests (CT and MRI), electroencephalogram and Electrocardiogram show no alterations

**Conclusions:**

Levetirazetam can cause psychiatric adverse effects. it is important to make a proper diagnosis before a first psychotic outbreak in later life. Drugs that can produce psychiatric side effects should be identified and patients should be inform.

**Disclosure:**

No significant relationships.

